# Lubricity and Rheological Properties of Highly Dispersed Graphite in Clay-Water-Based Drilling Fluids

**DOI:** 10.3390/ma15031083

**Published:** 2022-01-30

**Authors:** Quande Wang, Michal Slaný, Xuefan Gu, Zhipeng Miao, Weichao Du, Jie Zhang, Chen Gang

**Affiliations:** 1 State Key Laboratory of Petroleum Pollution Control, Xi’an Shiyou University, Xi’an 710065, China; 20212070871@stumail.xsyu.edu.cn (Q.W.); xuefangu@xsyu.edu.cn (X.G.); duweichao@xsyu.edu.cn (W.D.); zhangjie@xsyu.edu.cn (J.Z.); 2 Shaanxi Province Key Laboratory of Environmental Pollution Control and Reservoir Protection Technology of Oilfields, Xi’an Shiyou University, Xi’an 710065, China; 3 Institute of Construction and Architecture, Slovak Academy of Sciences, 845 03 Bratislava, Slovakia; 4 Xi’an Key Laboratory of Tight Oil (Shale Oil) Development, Xi’an Shiyou University, Xi’an 710065, China; miao0120@163.com

**Keywords:** surfactant, lubricity, clay, drilling fluids, bentonite, graphite–cement composites

## Abstract

Improving the tribological characteristics of water-based drilling fluids by adding graphene-based lubricants has garnered attention because of the potential for a range of inorganic-material-based additives at high temperature. In this study, we constructed a green and simple adsorption approach to prepare highly dispersed graphite using a cationic surfactant for graphite modification. The findings demonstrated that the prepared graphite was highly dispersed in water and had a low sedimentation rate and small contact angle in distilled water. The concentration dosage of cetyltrimethylammonium chloride (CTAC) on graphite was 0.02 g/g. We evaluated the performance of the modified graphite as a lubricated additive in water-based drilling through a rheological study and viscosity coefficient measurement. The results showed that the viscosity coefficient of drilling fluid with 0.05% modified graphite was reduced by 67% at 180 °C. We proved that the modified graphite can significantly improve the lubrication performance of drilling fluid. Furthermore, we revealed the lubrication mechanism by analyzing the chemical structural and crystalline and morphological features of graphite through a particle size test, zeta potential test, Fourier transform infrared (FTIR) spectroscopy, X-ray powder diffraction (XRD), and scanning electron microscopy (SEM) measurements. The results indicated that the modification of graphite by CTAC only occurs through physical adsorption, without changing the crystal structure. These findings provide a reference for the development of high-performance water-based drilling fluids.

## 1. Introduction

Drilling fluids are complex chemical systems that play critical roles in the petroleum industry [1,2]. Nowadays, polymers, especially eco-friendly biopolymers, are specifically added to drilling fluid to maintain its rheological properties, viscosity, filtration, and other characteristics [3,4]. Unfortunately, these polymer-based agents experience serious degradation and fail to retain their properties under harsh conditions such as high pressure and high temperature (HPHT) [5,6]. As such, drilling fluids need an additive with high stability. In addition, our research group has conducted research in the field of drilling fluid treatment agents [7,8], as a result of which we proposed some inorganic materials as desirable additives under HPHT conditions [9,10]. Graphite is an atomic crystal with carbon atoms connected by covalent single bonds to form a stable regular hexagonal network structure. Covalent single bonds are chemical bonds with very high bond energy and thus can only be destroyed by extremely high energy. For instance, graphite is oxidized by oxygen in the air when the temperature exceeds 600 °C. Therefore, graphite has strong resistance to high temperatures [11,12]. Graphite can also be used in graphite–cement composites. The key feature of this material is its conductive and porous microstructure that is created because of a synergic effect between the cementitious matrix and graphite particles.

Another issue with water-based drilling fluids during drilling works is their relatively high friction, causing drilling engineering problems in directional, horizontal, extended-reach, cluster, and ultra-deep wells [13]. In these diverse wellbore structures, the torque and friction force are higher during the drilling process. With the increasing difficulty of oil and gas exploration, complex wells such as extended-reach, horizontal, and ultra-deep wells are frequently used, where water-based drilling fluid is the main choice for drilling. Compared to water-based drilling fluid, oil-based drilling fluid produces the least friction and torque. However, with increasingly stringent environmental requirements, the environmental performance of drilling fluid has attracted much attention [14]. The main lubricants used in drilling fluid in this field are mineral oil and vegetable oil, which cause environmental pollution, and have been gradually replaced. Lubricating additives have high surface activity, and improve surface adhesion and lubricity [15,16]. Therefore, a water-based drilling fluid mud with lubricating additives as designed. This lubricating additive is environmentally friendly, cost-effective, and can provide similar lubricating effects as oil- and synthetic-based drilling fluids [17,18]. Graphene-based lubricants are considered water-based drilling fluid lubricant candidates [19]. Graphite has a hexagonal crystal structure, and the atoms in the crystal lattice are evenly distributed on parallel planes [20,21]. Since the bonding force between atoms in the same graphene layer is stronger than the bonding force between planes, shear easily occurs between layers. The friction between the layers is extremely low, and relative displacement between the layers can easily occur [22,23]. Therefore, if the graphite particles are attached on the interface, the tangential friction is small, which ensures that the graphite provides a suitable lubricating effect [24,25].

Dispersion stability is thought to be the key factor for graphene-based lubricants [26,27]. Graphene displays incompatibility with water owing to its intrinsic hydrophobicity [28], thus improving the dispersion of graphene in water is challenging. Modification of graphene by the self-assembly of a surfactant is a novel technological solution. In this study, we modified the surface of natural flake graphite by using a cationic surfactant CTAC for use as a lubricant in water-based drilling fluid, which can overcome the problem of natural flake graphite not evenly dispersing in drilling fluid due to its hydrophobicity. We only used the highly dispersed modified graphite described in this paper for a performance evaluation in a simple water-based drilling fluid, and its application in complex wells has certain limitations. Our findings provide some guidance for future research on the lubricity of water-based drilling fluid.

## 2. Experimental Materials and Methods

### 2.1. Materials and Reagents

Calcium and sodium-based bentonite were purchased from Fengyun Chemical Co., Ltd. (Xi’an, China). Sodium carbonate was obtained from Shengao Chemical Reagent Co., Ltd. (Tianjin, China). Graphite powder was purchased from Risheng Graphite Co., Ltd. (Qingdao, China) and cetyltrimethylammonium chloride (CTAC) was purchased from Aipuno Co., Ltd. (Qingdao, China).

### 2.2. Drilling Fluid Evaluation

These components were mixed well according to the following procedure: 0.7 g of sodium carbonate was added to tap water and stirred for 3 min. This step was repeated to dissolve 14 g calcium bentonite, and the mixture stirred at high speed for 2 h, and then aged for 24 h [29]. For the preparation of the treatment drilling fluid, the base drilling fluid and modified graphite were aged for 6 h, stirred at high speed for 10 min, and then its performance was tested [30]. The formula of the modified graphite slurry is shown in Table 1. The rheological, filtration, and lubrication properties of drilling fluid, including apparent viscosity (AV), plastic viscosity (PV), yield point (YP), API filtration (F_L_), and viscosity coefficient (VC) were obtained. We used a viscometer (ZNN-D6S, Hetongda Co., Ltd. Qingdao, China), a medium pressure filtration instrument (GJSS-B12K, Haitongda Co., Ltd. Qingdao, China), and a viscosity coefficient instrument (Qingdao Hetongda Co., Ltd. Qingdao, China) according to the Chinese National Standard GB/T 16783.1-2006.

### 2.3. Screening the Amount of CTAC

Cetyltrimethylammonium chloride (CTAC) and graphite in ratios of 0.005, 0.007, 0.010, 0.020, 0.025, 0.033, 0.050, and 0.1 g/g were placed in a flask. Subsequently, distilled water was added into the flask, and the mixture was stirred at 45 °C for 4 h. The mixture was centrifuged, the supernatant was removed, and the modified graphite was separated and dried at 60 °C over night. The absorbance and zeta potential of modified graphite were measured [30,31]. The modified graphite was characterized using a laser particle size measurement analyzer (SMA, Horiba, Japan), X-ray diffraction (JDX-3530, JEOL, Tokyo, Japan), FTIR spectroscopy (Thermo Electron Co., West Palm Beach, FL, USA), contact angle measurement (KRÜSS, Hamburg, Germany), and scanning electron microscopy (SEM, JSM-6390A, JEOL, Tokyo, Japan).

### 2.4. Infrared Spectroscopy

The dried modified graphite samples were ground. During the test, the ground sample was mixed with KBr in a ratio of 1:200, placed into a tablet press, and pressed into transparent flakes. FTIR measurements were performed in the range 4000–400 cm^−1^, and 64 scans at a resolution of 4 cm^−1^ were used for analysis. [32]. 

### 2.5. Contact Angle Measurement

The modified graphite powder was pressed into graphite flakes under a four-column press at a pressure of 20 MPa, and then the pressed flakes were placed in contact angles. The contact angle between the flakes and distilled water was measured on a contact angle measuring instrument to optimize the optimal dosage of CTAC on graphite [33].

### 2.6. Laser Particle Size Measurement

Dried modified graphite samples were used for particle size measurements in the laser particle size experiment to obtain the median and average particle sizes of bentonite particles in different drilling fluid treated with treatment agent. The change in bentonite particle size was analyzed according to these mesurements [34].

### 2.7. Zeta Potential Measurement

The zeta potential of the supernatant of the solution was measured on an omni multi-angle particle size and high-sensitivity zeta potential analyzer. The changes in the zeta potential of graphite with different dosages of adsorbent were analyzed [35].

### 2.8. X-ray Diffraction

The dried modified graphite sample was analyzed using a D8ADVAHCL X-ray diffractometer (Bruker, Berlin, Germany) with the following parameters: Cu target, ceramic X-ray tube, a tube current of 40 mA, a tube voltage of 40 kV, a step size of 0.02°, and a scanning range of 5–90°(2θ). The sample was found to be modified graphite, and the change in layer spacing of bentonite under different conditions was calculated by the Bragg equation (nλ = 2sinθ), and the change in graphite crystal structure was analyzed [36].

## 3. Results and Discussion

### 3.1. Graphite Sedimentation Experiment

**Figure 1:**
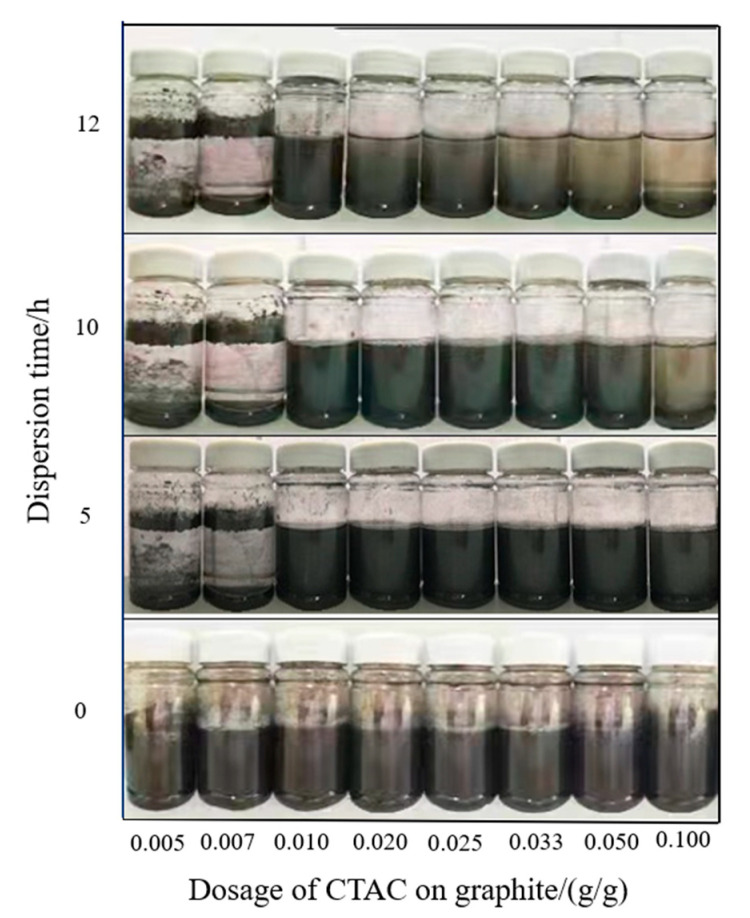
The effect of CTAC dosage on the dispersibility of graphite.

We first investigated the effect of CTAC dosage on the dispersibility of graphite, and the experiments were designed as shown in Table 2. The dispersion of graphite in distilled water was observed and recorded within a certain time, as shown in Figure 1. As shown in Figure 1, the unmodified graphite floated on the surface of distilled water, which showed obvious hydrophobic characteristics. The suspension also produced foam after shaking because of the foaming effect of the excess CTAC modifier. With the increase in the CTAC dosage, the foam increased gradually, and almost no foam was found in samples 1–4. The modified graphite of samples 3–8 homogeneously dispersed in water in this experiment, whereas the small dosage of graphite in samples 1 and 2 floated on the surface of the water. This showed that CTAC changed the surface of graphite from hydrophobic to hydrophilic; however, CTAC alone was not enough for samples 1 and 2, which still exhibited hydrophobicity.

Within 5 to 12 h, an amount of graphite still floated on the water surface of samples 1 and 2. In samples 3–8, with the increase in CTAC dosage, the degree of sedimentation gradually decreased. The modified graphite samples 3 and 4 showed excellent dispersion, and the modified graphite in samples 1 and 2 precipitated to the bottom of the bottle. Therefore, the dosage of CTAC was controlled at about 0.0200 g/g, which not only improved the hydrophilicity of graphite but also ensured the dispersion sustainability of graphite in solution.

### 3.2. Infrared Spectroscopy

**Figure 2:**
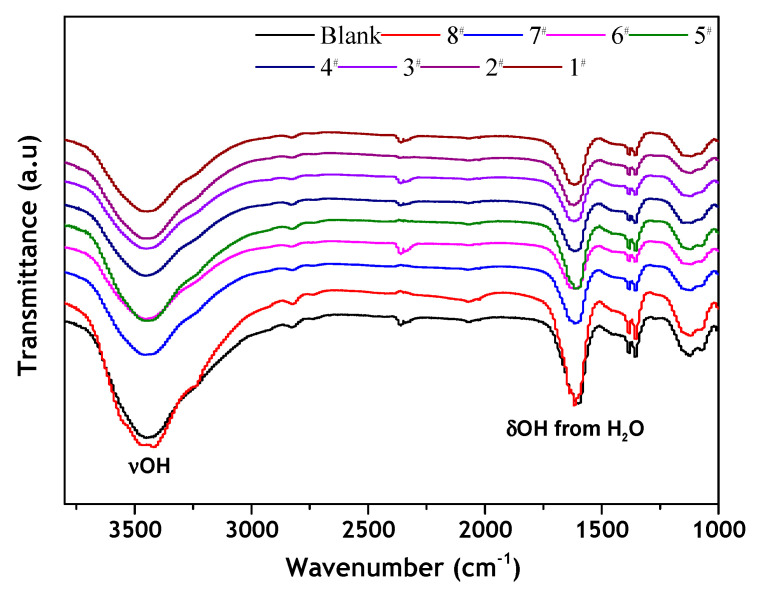
FTIR spectra of graphite before and after modification with different dosages of CTAC.

The infrared spectra of the blank and samples 1–8 were measured by Fourier transform infrared spectroscopy (FTIR). Figure 2 shows that the characteristic peaks in the infrared spectrum of the graphite modified with CTAC in samples 1–8 did not obviously change. This means that no chemical reaction occurred between the CTAC and graphite. However, the hydrophobicity/hydrophilicity of the measured samples changed. It is possible that the graphite changed from hydrophobic to hydrophilic due to physical adsorption on the graphite surface [37].

### 3.3. Contact Angle Measurement

**Figure 3:**
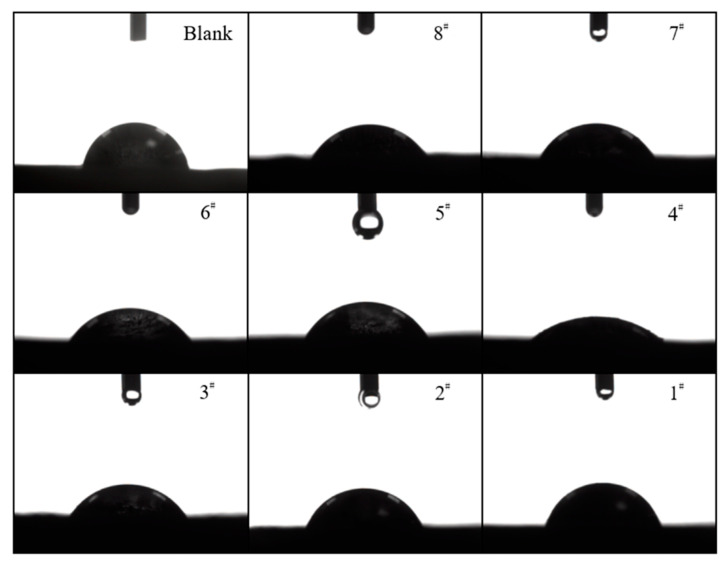
The contact angle between the graphite surface and distilled water before and after modification with CTAC.

The contact angle between the graphite and distilled water before and after the modification was measured using a contact angle measuring instrument. The contact angle was calculated by applying the angle measurement method. We used the average value of the contact angles on the left and right sides as the average contact angle. The results of the measurements are shown in Figure 3. The specific values are shown in Table 3. Figure 3 and Table 3 show that with the increase in the amount of CTAC, the contact angle between graphite flakes and distilled water roughly decreased first and then increased. The average contact angle between graphite flake and distilled water was 80.25°. Compared to untreated graphite, the average contact angles between graphite flakes of samples 1–8 and distilled water decreased by: 8.00°, 14.5°, 24.25°, 43.25°, 20.50°, 24.00°, 19.50°, and 21.75°, respectively. The average contact angle between the sample 4 graphite flakes and distilled water was the smallest when the dosage of CTAC on graphite was 0.020 g/g, which is consistent with the experimental results of graphite sedimentation described above.

### 3.4. Particle Size Measurement

The particle sizes of the blank and samples 1–8 were measured using a laser diffraction particle size analyzer. Table 4 shows that the average particle size of modified graphite reduced from 102.80 μm to 25.11, 17.10, 15.45, 10.03, 11.89, 15.85, 17.77, and 22.04 μm for samples 1–8, respectively. The median particle size reduced from 90.25 μm to 18.98, 13.71, 12.54, 7.93, 9.53, 12.57, 14.60, and 19.68 μm, respectively, compared to unmodified graphite. With the increase in the amount of CTAC, the average and median particle sizes of graphite decreased first and then increased, which showed that an excessive amount of CTAC leads to coagulation of graphite, resulting in a poor dispersion effect between graphite particles. Too little CTAC will cause incomplete adsorption on the surface of graphite. The experimental results showed that when the dosage of CTAC on graphite was 0.0200 g/g, the average and median particle sizes of graphite were the smallest, which is consistent with the best dispersion. Therefore, the best dosage of CTAC on graphite as found to be 0.0200 g/g.

### 3.5. Zeta Potential Measurement

The suspension of the blank and samples 1–8 was measured using an omni multi-angle particle size and high-sensitivity zeta potential analyzer to analyze the zeta potential of graphite before and after modification with different dosages of CTAC. Table 5 shows that zeta potential of the modified graphite increased from 8.65 mV to 30.77, 35.05, 35.49, 40.86, 50.25, 50.19, 59.71, and 65.30 mV for samples 1–8, respectively. We found that the adsorption of CTAC increased the zeta potential of graphite. This change in potential indicated that the stability of the formed suspension was enhanced.

### 3.6. X-ray Diffraction Analysis

**Figure 4:**
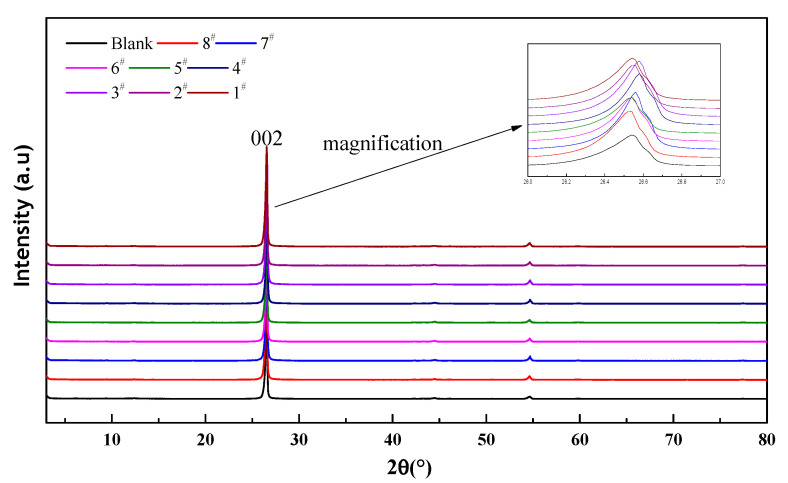
X-ray diffraction patterns of modified graphite.

X-ray diffraction experiments were conducted on the blank and samples 1–8 with an X-ray polycrystalline diffractometer to analyze the change in their crystal structure. The XRD pattern in Figure 4 shows that the characteristic peak of modified graphite did not significantly change. This means that the adsorption of CTAC does not change the crystal structure of graphite; in other words, the modification does not affect the interlayer friction coefficient of graphite.

### 3.7. Scanning Electron Microscopy

**Figure 5:**
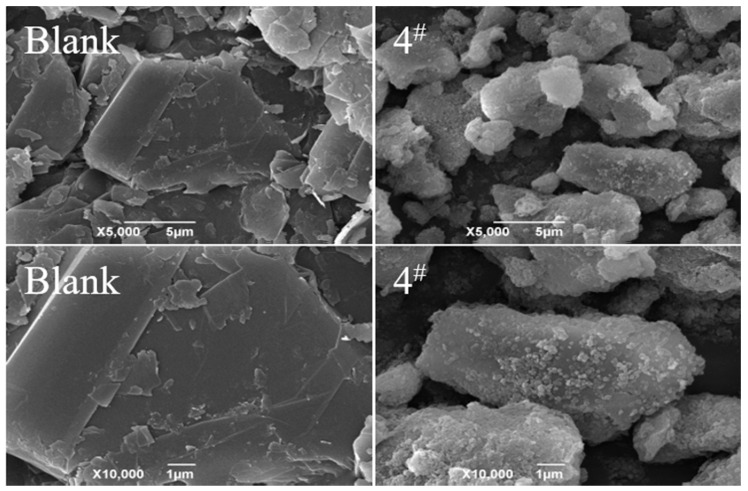
SEM image of the graphite surface before (above) and after (below) modification with CTAC.

SEM was used to study the morphology of modified graphite; the sample 4 graphite was selected as an example. Figure 5 shows that the size of the modified graphite particles significantly reduced, which means the modification increased the dispersion of the graphite flakes and reduced the aggregation. The smaller the graphite particles, the better the dispersion of graphite in aqueous solutions [38,39,40]. This result is completely consistent with the results in the previous experiments.

### 3.8. Performance in Drilling Fluid

Through the experiments above, we selected sample 4 as the best sample for use as a lubricant in an experiment with a water-based drilling fluid under different temperatures. Table 6 shows that the modified graphite had a certain lubricating effect at 180 °C, but it lost its lubricating effect at 190 °C. At almost every temperature, the lubricity increased with the increase in dosage. However, increasing the dosage worsens the lubricity, so an appropriate dosage must be applied.

**Figure 6:**
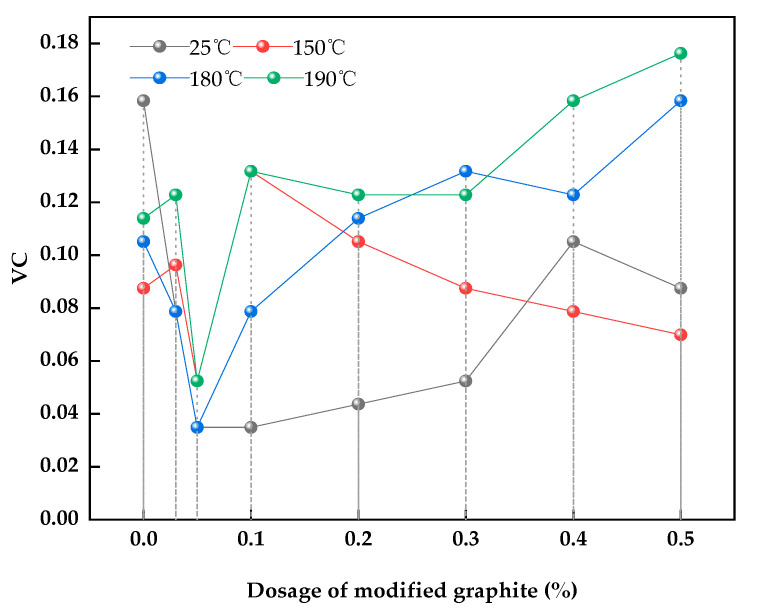
Relationship between the addition of different dosages of modified graphite and the viscosity coefficient.

Figure 6 shows that when the dosage of modified graphite was 0.05%, the viscosity coefficient of drilling fluid decreased from 0.1051 to 0.0349 at 180 °C, which is a decrease of 67%.

**Figure 7:**
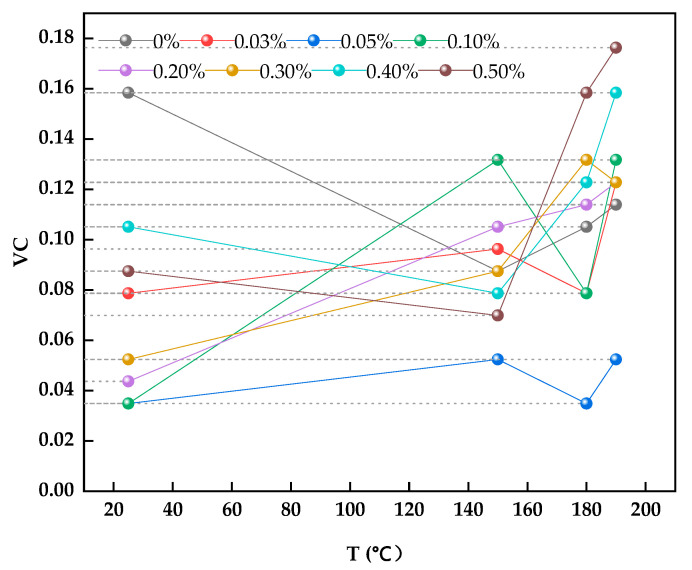
Relationship between the viscosity coefficient and temperature of the drilling fluid with different dosages of modified graphite.

Figure 7 shows that the dosage of modified graphite of 0.05% had good lubricity to the drilling fluid, and at this dosage, the minimum viscosity coefficient was achieved at 180 °C. Table 5 shows that with 0.05% modified graphite, the viscosity coefficient (VC) of drilling fluid reduced from 0.1584 to 0.0349. At 150 °C, the VC of drilling fluid decreased from 0.0875 to 0.0524. The VC is decreased from 0.1051 to 0.0349 at 180 °C. Notably, the addition of modified graphite had no obvious effect on the other parameters.

### 3.9. Mechanism

**Figure 8:**
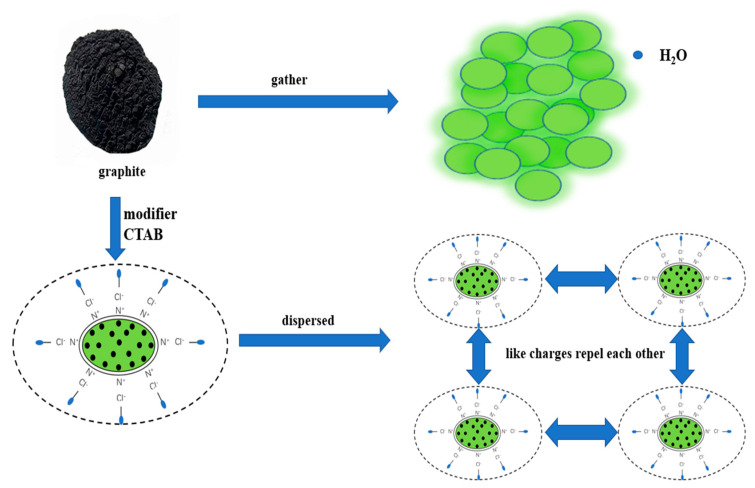
Mechanism of adsorption and dispersion of graphite.

From the results above, we found that the original hydrophobicity of graphite can be changed to hydrophilicity by the adsorption of CTAC on its surface. The group on CTAC is hydrophobic, so can adsorb on the surface of graphite. As a result, the graphite surface was covered by a layer of amino groups with positive charges and a diffusion layer of chloride, as shown in Figure 8. Due to the formation of diffusion electric double layers, this modified graphite shows repulsion with the same charge, which can reduce the particle size of graphite particles. This change can enhance the dispersion of graphite in aqueous solution or drilling fluid, and then enhance the lubrication performance of graphite. In addition, the method of adsorption of CTAC on graphite is physical adsorption, which is strongly affected by temperature. Therefore, CTAC may desorb under high temperatures, resulting in re-aggregation of modified graphite and the reduction in lubrication.

## 4. Conclusions

In this study, we modified graphite with CTAC using a simple method. Based on the FTIR, XRD, and SEM characterization, we found that the modification of graphite was achieved by physical adsorption, and its crystal structure did not change; only its dispersion was enhanced. The modified graphite was evaluated in a water-based drilling fluid, and the results showed that, when the amount of modified graphite was 0.05%, the viscosity coefficient at 180 °C decreased from 0.1051 to 0.0349, and the modified graphite showed a certain lubricating effect at this temperature. However, this effect was lost at 190 °C. At almost every temperature, the lubricity increased with the increase in dosage. The absorption of CTAC can form diffusion electric double layers and enhance the repulsion between particles. This change enhances the dispersion of graphite in an aqueous solution or drilling fluid and enhances the lubrication performance of graphite.

## Figures and Tables

**Table 1:** Function of the modified graphite slurry.

Component	Function	Type	Value
Tap water (mL)	Fluid base	-	350
Calcium bentonite (g)	Drilling mud	Industrial grade	14
Sodium carbonate (g)	Hardness control	Analytical purity	0.7
CTAC (g)	Modification	Analytical purity	-
Graphite (g)	Lubrication	Industrial grade	-

**Table 2:** Graphite sedimentation experiment.

Sample No.	Graphite (g)	CTAC (g)	Dosage of CTAC on Graphite (g/g)
1	4.00	0.020	0.005
2	4.00	0.027	0.007
3	4.00	0.040	0.010
4	4.00	0.080	0.020
5	4.00	0.100	0.025
6	4.00	0.130	0.033
7	4.00	0.200	0.050
8	4.00	0.400	0.100

**Table 3:** Measured contact angles between distilled water on the graphite surface before and after modification with CTAC.

Sample No.	Left Contact Angle Value (°)	Right Contact Angle Value (°)	Mean Contact Angle (°)
1	80.50	80.00	80.25
2	72.50	72.00	72.25
3	66.00	65.50	65.75
4	55.00	57.00	56.00
5	36.50	37.50	37.00
6	50.00	59.50	59.75
7	56.50	56.00	56.25
8	62.50	59.00	60.75

**Table 4:** Average and median particle sizes of graphite before and after modification with CTAC.

Sample No.	The Average Particle Size (μm)	Median Particle Size (μm)
Blank	102.80	90.25
1	25.11	18.98
2	17.10	13.71
3	15.45	12.54
4	10.03	7.93
5	11.89	9.53
6	15.85	12.57
7	17.77	14.60
8	22.04	19.68

**Table 5:** Zeta potential of graphite before and after modification with CTAC.

Sample No.	Zeta Potential Value (mV)
Blank	8.65
1	30.77
2	35.05
3	35.49
4	40.86
5	50.25
6	50.19
7	59.71
8	65.30

**Table 6:** Performance of drilling fluid with the addition of different dosages of CTAC-modified graphite.

Temperature (°C)	Dosage of Modified Graphite (%)	PV (mPa·s)	YP (Pa)	AV (mPa·s)	F_L_ (mL)	VC
25	0	3.00	0.20	3.00	16.5	0.1584
	0.03	3.00	0.20	3.00	16.2	0.0787
	0.05	2.50	0.50	3.00	16.4	0.0349
	0.10	2.50	0.50	3.00	15.8	0.0349
	0.20	3.00	0.25	3.25	15.5	0.0437
	0.30	2.50	0.50	3.00	16.0	0.0524
	0.40	2.50	0.50	3.00	15.0	0.1051
	0.50	1.50	1.25	2.75	16.0	0.0875
150	0	3.00	0.25	3.25	18.4	0.0875
	0.03	3.00	0.20	3.00	23.4	0.0963
	0.05	3.00	0.20	3.00	21.9	0.0524
	0.10	4.00	0.25	4.25	26.5	0.1317
	0.20	3.00	0.25	3.50	22.5	0.1051
	0.30	3.50	0.50	4.00	24.5	0.0875
	0.40	3.50	0.25	3.75	25.0	0.0787
	0.50	2.50	0.50	3.00	18.0	0.0699
180	0	3.00	0.20	3.00	20.1	0.1051
	0.03	2.50	0.25	2.75	25.6	0.0787
	0.05	2.50	0.25	2.75	24.4	0.0349
	0.10	3.50	0.50	4.00	32.0	0.0787
	0.20	3.00	0.25	3.25	32.5	0.1139
	0.30	4.00	0.25	4.25	41.5	0.1317
	0.40	3.00	0.25	3.25	30.5	0.1228
	0.50	3.50	0.50	4.00	28.5	0.1584
190	0	3.25	0.25	3.50	22.3	0.1139
	0.03	3.00	0.25	3.00	28.2	0.1228
	0.05	2.50	0.50	3.00	27.5	0.0524
	0.10	3.50	0.50	3.50	31.5	0.1317
	0.20	3.50	0.25	3.75	29.6	0.1228
	0.30	3.00	0.75	3.75	32.5	0.1228
	0.40	3.00	0.25	3.00	27.0	0.1584
	0.50	3.00	0.50	3.50	29.5	0.1763

## Data Availability

The data presented in this study are available from the corresponding author upon request.
